# Long‐lasting effects of logging on beetles in hollow oaks

**DOI:** 10.1002/ece3.4486

**Published:** 2018-09-27

**Authors:** Hanne E. Pilskog, Anne Sverdrup‐Thygeson, Marianne Evju, Erik Framstad, Tone Birkemoe

**Affiliations:** ^1^ Department of Ecology and Natural Resource Management Norwegian University of Life Sciences Aas Norway; ^2^ Norwegian Institute for Nature Research Oslo Norway

**Keywords:** Coleoptera, extinction debt, historical logging, *Quercus*, saproxylic, spatial scales, temporal scale, veteran tree

## Abstract

There is growing evidence that biodiversity is important for ecosystem functions. Thus, identification of habitat requirements essential for current species richness and abundance to persist is crucial. Hollow oaks (*Quercus* spp.) are biodiversity hot spots for deadwood‐dependent insect species, and the main objective of this paper was to test the effect of habitat history and current habitat distribution at various spatial scales on the associated beetle community. We used a gradient spanning 40 km from the coast to inland areas reflecting historical logging intensity (later and lower intensities inland) through 500 years in Southern Norway, to investigate whether the historical variation in oak density is influencing the structure of beetle communities in hollow oaks today. We trapped beetles in 32 hollow oaks along this gradient in forested and seminatural landscapes over two summers. We found higher species richness and total abundance inland consistent with our expectation based on historic logging intensity. Scale‐specific environmental variables also affected the response; beetle abundances were controlled by local conditions, whereas beetle species richness responded to habitat on the landscape scale. This indicates that long time continuity as well as large areas of favorable habitat is necessary to maintain beetle species richness through time in these highly long‐lasting structures.

## 1 INTRODUCTION

Habitat loss is currently one of the greatest threats to biodiversity and ecosystems worldwide (Millennium Ecosystem Assessment, 2005; Sala et al., 2000), with species going extinct at a rate that suggests we are entering a sixth mass extinction (Barnosky et al., 2011). The increasing number of studies indicating that species richness is important for ecosystem functions makes this decline particularly worrying (Cardinale et al., 2012; Tilman, Isbell, & Cowles, 2014). To counteract the extinction trend and maintain ecosystem functions, efficient conservation measures are needed. Thus, identification of habitat requirements essential for current biodiversity to persist is crucial.

Species richness and composition are affected by processes at several spatial scales (Cornell & Harrison, 2014; Jackson & Fahrig, 2015; Wiens, 1989). For example, at a regional scale, climate may control a species’ distribution, but at a local scale, biological processes such as competition can override the climatic effects (Wiens, 1989), making climate a poor predictor of a species’ local occurrence. The responses to spatial scales are likely to be species dependent (Sverdrup‐Thygeson, Gustafsson, & Kouki, 2014; Wiens, 1989), and spatial studies of communities face two major challenges: Firstly, the relevant species‐specific scales are rarely known, and secondly, a community will normally contain species with a range of spatial responses (Holland, Bert, & Fahrig, 2004; Jackson & Fahrig, 2015). One way forward can be to categorize species that are likely to have similar spatial responses (Dupré & Ehrlén, 2002; Henle, Davies, Kleyer, Margules, & Settele, 2004; Sverdrup‐Thygeson, Bendiksen, Birkemoe, & Larsson, 2014). Finding shared scales of responses for species aggregates is also useful for conservation purposes as it might enable correct management recommendations (Bergman, Jansson, Claesson, Palmer, & Milberg, 2012). Whatever approach taken, in order to reveal important scale‐dependent ecological patterns within a community, the inclusion of multiple scales is needed (Jackson & Fahrig, 2015; Lindenmayer, 2000; Wiens, 1989).

All species communities change through time and are affected by past immigrations, extinctions, and fluctuation in environmental factors (Magurran & McGill, 2011). Several recent studies also show that local and regional habitat loss history can have substantial impact on current communities (Helm, Hanski, & Pärtel, 2006; Kuussaari et al., 2009; Sverdrup‐Thygeson, Gustafsson, et al., 2014). If populations are not in equilibrium with their surroundings due to changes in the past, species can still be expected to go extinct locally even if habitat loss is halted (extinction debt) (Kuussaari et al., 2009). For example, the number of specialist plant species occurring in the calcareous grasslands of Estonia cannot be explained by current habitat area or connectivity, but by that present 70 years previously, before the subsequent massive loss of habitat (Helm et al., 2006). An estimated 40% of species in the remaining grassland could yet go extinct, a legacy of this past loss. Recording species number without considering past events therefore risks overestimating long‐term species richness and underestimating the threat of extinction (Helm et al., 2006). Despite their limited number, current studies of plants, lichens, insects, fish, and birds indicate that extinction debt is more common than previously recognized (Kuussaari et al., 2009).

Whereas the risk of species extinctions following habitat destruction is relatively well known, the effect on overall abundances within the same communities is less clear. Obviously, species at risk are likely to decline, but less‐sensitive species might potentially increase in number as a response to decreased competition. Opposing this pattern, Gonzalez and Chaneton (2002) demonstrated a decline in overall abundance and biomass in springtails following experimental habitat fragmentation. This effect was delayed relative to the decline in species richness.

In this study, we investigate how habitat history and current habitat distribution at different spatial scales affect the richness and abundance of species groups exhibiting varying degrees of habitat specialization. We studied beetles dependent on deadwood (saproxylic beetles) living in hollow oaks (*Quercus* spp.) and ask whether the history of forest exploitation influences present patterns of species richness and abundance, beyond what can be explained by the properties of individual oak trees, their close surroundings, and the wider landscape.

**Figure 1:**
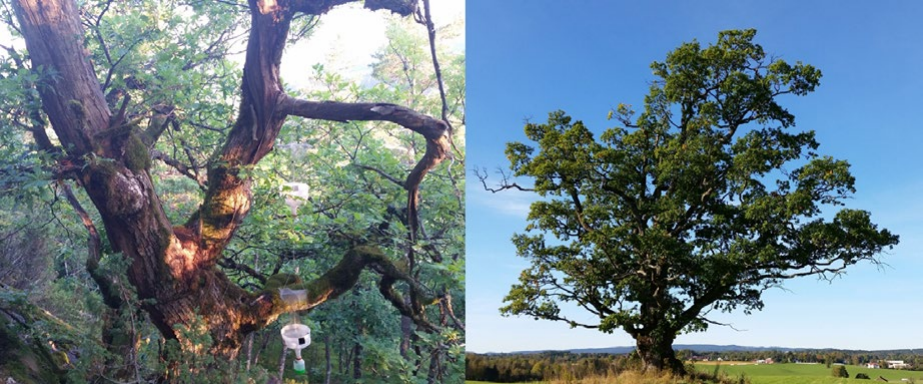
Veteran oaks (*Quercus* sp.) in forest and agricultural landscape. Window traps to collect insects are shown to the left

Veteran trees, with or without hollows, have been recognized as biodiversity hot spots, rich in rare and red‐listed species (Bütler, Lachat, Larrieu, & Paillet, 2013; Sverdrup‐Thygeson, 2009) (Figure 1). They provide ecological continuity through time and are keystone structures in many landscapes (Manning, Fischer, & Lindenmayer, 2006). However, veteran trees are often locally rare, occur in fragmented landscapes, and are declining globally (Gibbons et al., 2008; Lindenmayer, Laurance, & Franklin, 2012; Lindenmayer et al., 2014; Siitonen & Ranius, 2015). Veteran oaks are one of the most important environments for saproxylic species in Northern Europe (Hultengren, Pleijel, & Holmer, 1997; Siitonen & Ranius, 2015) and form a long‐lasting habitat for associated species (Nordén et al., 2014; Ranius, Niklasson, & Berg, 2009). As the oaks age, a range of microhabitats develop that are not present in younger trees, such as coarse bark, dead branches, and cavities with wood mold (Bütler et al., 2013; Siitonen & Ranius, 2015). As the cavities are created with the help of wood‐decaying fungi and insects, the wood mold accumulates in the cavities and consists of decaying wood and fungi that typically mix with remnants from bird nests, bird or bat droppings, dead insects, and other detritus creating a specialized habitat for many species (Sverdrup‐Thygeson, 2009). This process takes centuries, as most oaks start to develop cavities around 200 years of age (Ranius et al., 2009).

The hollow oaks in our study system have a fragmented distribution due to historical large‐scale logging of oak, and it is possible that the associated beetles are responding both to historical and current habitat density. To investigate whether the historical variation in oak density is important for beetles in hollow oaks, we used a gradient spanning 40 km from the coast to the inland reflecting historical logging intensity through 500 years. As large‐scale logging started earlier and was more intensive along the coast than in inland areas, the remaining hollow oaks along the coast are expected to have been isolated from other hollow oaks for longer than those inland. To assess the importance of current habitat and surroundings, we also included environmental variables at three spatial scales: the individual tree; its immediate surroundings (~30 m radius); and the landscape (~2 km radius).

We predict that (a) the richness and abundance of saproxylic oak‐beetle species will be lower close to the coast than at inland sites, reflecting the inferred difference in logging intensity and duration with distance from the coast; (b) the effect described in (a) is stronger for species most dependent on oak (mainly oak species) than for those with broader habitat preferences (broadleaf species and generalists); and (c) the effect of historical land use will be modified by scale‐specific environmental variables.

## 2 METHODS

### 2.1 Study area and design

#### 2.1.1 The logging of oak in Norway

Historically, Norway had large oak forests in Southern Norway growing right down to the coast (Vevstad, 1998; Vogt, 1886). The shortage of oak timber in Europe combined with the introduction of river sawmills in the 1520s set the scene for large‐scale logging and export of oak (Central Bureau of Statistics of Norway, 1977; Moore, 2010). Transporting the timber was the most demanding part of the trade. River transport (log floating) was difficult, could take several years, and led to substantial timber loss (Vevstad, 1998). Therefore, the easily accessible coastal areas were logged first (Vevstad, 1998). Oak was heavily harvested there from the 1520s, and throughout the 1600s, but by the end of the 17th‐century little oak suitable for logging was left (Central Bureau of Statistics of Norway, 1977; Moore, 2010; Vevstad, 1998). Already in the 1630s, many places along the coast lacked suitable oak timber (Tvethe, 1852), and it is safe to assume that logging of oak in general occurred inland from the mid‐17th century and onwards. As the number of mature oaks diminished, the logging for pine and spruce escalated and replaced oak as the most important timber trees (Vevstad, 1998). Oak never regained its dominance, even though the timber was highly valued as shipbuilding material until the late 19th century. For more information about the history of oak logging see Supporting Information Appendix S1.

#### 2.1.2 Study areas

**Figure 2:**
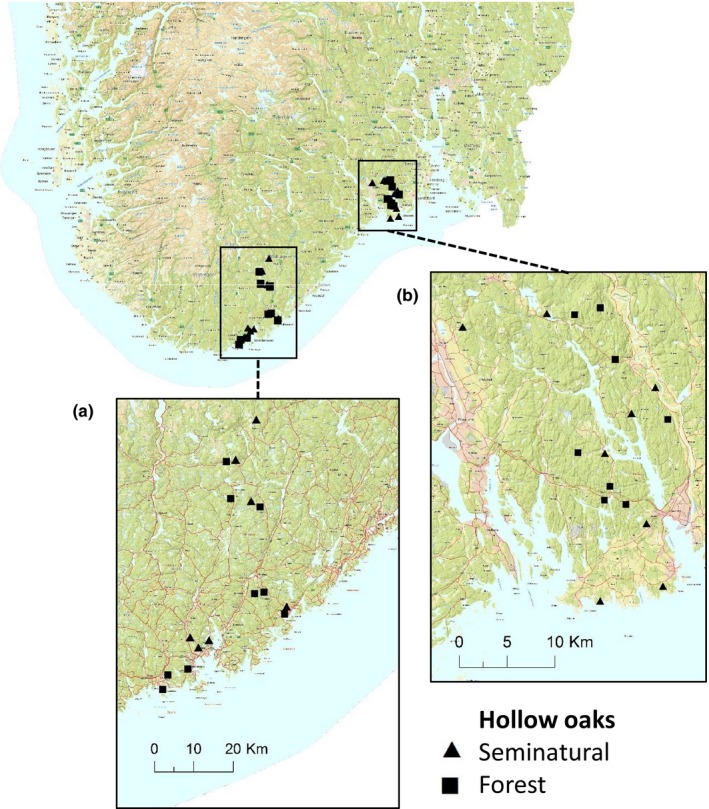
Locations of the sampled hollow oaks (*n* = 32) along the coast–inland gradient in Southern Norway. The hollow oaks were situated in forests and seminatural landscapes (squares and triangles) in the Agder (a) and Larvik (b) regions

To study a geographical gradient representing historical logging intensity and duration, we selected hollow oaks *Quercus robur* and *Quercus petraea* along a coast–inland gradient in two regions, Agder and Larvik, in Southern Norway. Agder is situated in the south, with hollow oaks from the coast to 40 km inland, while Larvik is located in the southeast with hollow oaks from the coast to 25 km inland (Figure 2).

The two sampling regions are both within the main area of oak distribution in Norway and span the nemoral, boreonemoral, and southern boreal vegetation zones (Moen, 1999). In Agder (Vest‐ and Aust‐Agder counties), the forests are dominated by pine *Pinus sylvestris* (45%–53% of the forest), spruce *Picea abies* (20%–24%), and deciduous trees (16%–29%) (Tomter & Eriksen, 2001; Tomter, Eriksen, & Aalde, 2001). Around 8% of the productive forest volume in the Agder region today is oak. Larvik is part of Vestfold county, where forests are dominated by spruce (45%), deciduous trees (35%), and pine (15%) (Eriksen, Tomter, & Ludahl, 2006). Only 2.7% of the productive forest volume is oak, but there is a higher percentage of large trees (9.5% with trunk diameters > 45 cm) compared with 1%–4% in Agder (Eriksen et al., 2006; Tomter & Eriksen, 2001; Tomter et al., 2001).

We sampled 16 hollow oaks in each region with a minimum distance of 1.5 km between each to ensure independent sampling. We selected individuals with a visible hollow above ground and the presence of wood mold. As the species composition of beetles in hollow oaks varies between forest trees and those in agricultural or urban landscapes (Skarpaas, Diserud, Sverdrup‐Thygeson, & Ødegaard, 2011; Sverdrup‐Thygeson, Skarpaas, & Ødegaard, 2010), we avoided the most culturally influenced trees, such as heavily pollarded trees in parks or cities and wide‐branched solitary trees in open landscapes. Our study included trees in forest (*n* = 17) and seminatural habitats (*n* = 15). The latter represents oaks in forest edges along fields or close to settlements. The seminatural and forest oaks were evenly distributed along the gradient and between the regions (Figure 2, Table 1). We did not differentiate between *Q. robur* and *Q*.* petraea* as this is unlikely to affect the beetles.

**Table 1:** Predictor variables included in the statistical analyses (variables in italics were not included in the model selection due to collinearity with other variables)

Scale	Name	Units or categories	Explanation
Tree	Circumference	cm	Circumference measured at breast height (1.3 m above ground) (min. 80, mean 228, max. 500)
	Tree form	low, middle, high	The shape of the tree was categorized based on the position of the tree crown into low (*n* = 8 trees), middle (*n* = 16), or high (*n* = 8) position. The growth form is a combination of current and past growing conditions
Local	Forest density	basal area (m^2^/ha)	Forest density was measured as the basal area of trees around the hollow oak using a relascope with 1‐cm opening (min. 5, mean 16.6, max. 36)
	Landscape	forest, seminatural	Oaks were situated either in forests (*n* = 17) or in seminatural habitats (*n* = 15). Both types were evenly distributed in the regions and along the coast–inland gradient (forest sites: Agder: *n* = 7, Larvik: *n* = 8; seminatural: Agder: *n* = 9, Larvik: *n* = 8)
	Oaks	oak trees	Number of oak trees ≥20 cm in diameter at breast height within 42 × 42 m square around the oak (min. 0, mean 12.2, max. 32)
	Hollow oaks	hollow oaks	Number of hollow oaks ≥20 cm in diameter at breast height, within 42 × 42 m square around the oak (min. 0, mean 1.9, max. 6)
	Deadwood	m^3^/ha	Minimum volume of deadwood within a 42 × 42 m square around the oak. Standing and lying deadwood ≥1 m in length was counted in size classes: small (diameter: 10–20 cm), medium (21–40 cm), and large (>40 cm), and minimum deadwood volume was estimated based on the smallest diameter in each size class (min. 0.039, mean 0.466, max. 1.172)
Landscape	Favorable habitat	ha	Area of favorable habitat measured in hectare within 2 km radius of the hollow oaks. See the main text for more details (min. 0.17, mean 3.00, max. 11.17)
	Deciduous forest	ha	Area covered by deciduous‐dominated forest within 2 km of the hollow oaks. Deciduous dominated was defined as >50% of the volume being deciduous trees (min. 19.53, mean 175.23, max. 412.87)
	*Forest cover*	ha	Area covered by forest within 2 km of the hollow oaks (min. 173.24, mean 780.45, max. 1119.59)
	*Old forest*	ha	Area of old forest (average age > 80 years) within 2 km of the hollow oaks (min. 3.15, mean 131.28, max. 412.72)
	*Forest volume*	m^3^/ha	Average forest volume (measured without bark) per hectare within 2 km of the hollow oaks (min. 61.74, mean 99.26, max. 126.30)
Coast–inland gradient	Distance to coast	km	Shortest distance to the coast measured as a straight line, used as a proxy for historical logging intensity and duration. For some sites, a straight line to the likely destination was used as the shortest line did not reflect the probable transport route of timber due to difficult terrain (min. 0.04, mean 12.89, max. 40.47)
	Precipitation	mm	Sum of average precipitation in the four warmest months (June–September) for the period 1961–1990 (min. 338, mean 411, max. 518)
	*Temperature*	°C	Average summer temperature in the four warmest months for the period 1961–1990 (min. 11.7, mean 13.2, max. 14.3)

#### 2.1.3 The coast–inland gradient

Distance to coast was measured on a regional scale along the coast–inland gradient and was used as a proxy for how accessible and attractive the oaks were for historical logging. Oaks close to the coast were assumed to be isolated earlier and exposed to generally higher land‐use pressures. The shortest distance from the oaks to the coast was measured as a straight line (Euclidean distance) using ArcMap 10.2.2 (Table 1). In the Larvik region, a straight line to the known destination for logged timber (Larvik city) was used for four trees as the shortest distance to the coast represented an impossible transport route for timber because of the terrain.

Because climate is likely to vary along the coast–inland gradient, climate variables were included to separate the effects of climate and historical logging. We characterized each site by its mean summer temperature (°C) and total precipitation (mm) in the four warmest months (June–September). We used interpolated data from a 1 × 1 km^2^ grid made available by the Norwegian Meteorological Institute (see http://met.no/) for the period 1961–1990, assuming this to be representative of the climatic conditions prevailing in the study area (Table 1).

#### 2.1.4 Spatial scales

We characterized habitat quality at three spatial scales. The smallest spatial scale used was the *tree scale*. For each oak, we recorded the circumference at breast height (cm) and categorized the growth form of the tree (Table 1). The close surroundings were used to characterize the *local scale*. At each site, we counted the total number of oaks, number of hollow oaks (few or no other tree species possessed hollows), and the downed and standing deadwood of all tree species in different size classes in an area of 42 × 42 m^2^ around the oak (see Table 1). The square was defined by walking 30 m away from the focal oak in the cardinal directions (N, S, E, W) with the ending points forming the corners of the square. As a measure of the openness around the sampled oaks, we estimated forest density using stand basal area (m^2^/ha), measured through a relascope with a 1‐cm wide opening.

To characterize the surroundings of each sampled oak on a *landscape scale*, we included forest variables and a measure of favorable habitat in a 2 km radius, as this scale has proved to be important for species richness of saproxylic beetles (Bergman et al., 2012; Jacobsen, Sverdrup‐Thygeson, & Birkemoe, 2015). For the forest variables, we obtained information on forest cover and structure from satellite images of the landscape provided by the Norwegian Institute of Bioeconomy Research (NIBIO, 2016). ArcMap 10.2.2 was used to extract information on the 2‐km scale around the oak using the clip function, and we used information on forest cover, volume per hectare, area of deciduous trees, and cover of old forest (average tree age > 80 years old) (Table 1). As the forest today is dominated by spruce and pine, the “forest age” variable is unlikely to represent differences in historical logging of oak.

To include a measure of favorable habitat on the landscape scale, we used information from the Norwegian database for habitats (Naturbase) (Norwegian Environment Agency, 2015) on occurrences of hollow and large oaks (recorded as points registrations or polygons), hollow deciduous trees (point records), and standing and downed deadwood (recorded in polygons). In Larvik, we also included supplementary records of woodland key habitats relevant for oak‐associated saproxylics (Franc, Götmark, Økland, Nordén, & Paltto, 2007; Skoger, 2016; Götmark, Asegard, & Franc, 2011). Polygons without estimates were measured in ArcMap 10.2.2, and all records checked for overlap. To create a single habitat variable, we needed to convert all the records to a common scale. We therefore combined the point registrations of hollow and large trees within 2 km of the hollow oak with an estimated number of old oaks in the polygons. The number of single trees was then converted to a common scale of 30 trees/ha and merged with the data from the deadwood polygons (defined as minimum 20–40 trees/ha) (Baumann et al., 2001). As the woodland key habitats in Larvik are large and contain other nature types than only old oaks and deadwood, a conversion factor of 0.1 was used before adding the information from these polygons to the same variable (Table 1, see Supporting Information Appendix S3 for more details on the habitat variable). Because not all areas were completely mapped, we acknowledge that our “favorable habitat” variable could be underestimated in some areas.

#### 2.1.5 Insect sampling

Each oak was sampled for insects by a standard method used in previous studies (Sverdrup‐Thygeson, 2009; Sverdrup‐Thygeson et al., 2010): two flight interception traps (window size 20 × 40 cm^2^) for each oak, one in front of the cavity opening and one in the canopy (Figure 1). The insect traps were active from mid‐May to mid‐August in 2013 and 2014 and emptied once a month. We used a solution of propylene glycol, water, and liquid dish detergent in the collecting containers. The insects were transferred to a 7:3 mix of propylene glycol and ethanol and stored at −20°C until identification. The data from the two traps were pooled prior to the statistical analysis.

All beetles were identified to species and categorized according to their association with oaks (Supporting Information Appendix S2). Only saproxylic species associated with oak were included in our analyses. We used the following categories: “mainly oak” for species mainly occurring in oak; “broadleaf species” for species occurring only in oak and broadleaved trees; and “generalists” for species occurring in both oak and coniferous trees (Supporting Information Appendix S2).

### 2.2 Statistics

All statistical analyses were carried out in R. v. 3.1.0. To investigate whether the recorded environmental variables varied systematically along the coast–inland gradient, we calculated the correlation coefficients (Pearson's *r*) between the assorted site variables and distance to coast. We wanted to reduce the number of predictor variables prior to model selection, and therefore tested for collinearity and eliminated variables until variance inflation factors were below three, as recommended by Zuur, Ieno, Walker, Saveliev, and Smith (2009). Temperature was correlated with precipitation and distance to coast, and most of the forest variables covaried with distance to coast and with each other (excluded collinear variables shown in italics in Table 1).

We tested whether our two study regions, Agder and Larvik, should be included as random variables in the models by comparing generalized least square (GLS) and linear mixed‐effect (LME) models. We included all the variables in the GLS and LME models and compared their Akaike information criterion (AIC) scores. The GLS models generally had lower AIC values, and we proceeded without random effects, using generalized linear models (GLMs) with a Poisson distribution and log‐link function. For backward elimination, we used the drop1 function to find the optimal models based on AIC scores. The abundance data and species richness of “all species” and “oak generalists” were overdispersed, so we applied a negative binomial GLM using the glm.nb function from the MASS library in R, and stepAIC, to find the optimal models. When two models had almost identical AIC values (<1), we chose the simplest model. The optimal models were then tested against null models in analyses of deviance (for Poisson GLMs) or log‐likelihood tests (for negative binomial GLMs). An outlier caused substantial overdispersion (dispersion parameter > 1.3) in the overall and the oak generalist abundances. The outlier resulted from high numbers of the ant‐associated oak generalist *Haploglossa villosula* (Päivinen, Ahlroth, & Kaitala, 2002) in one tree, probably caused by a nest of the ant *Lasius fuliginosus*. *H. villosula* was present in most oaks (*n *=* *27) and was excluded from the abundance data to remove overdispersion and improve the diagnostic plots.

To investigate whether the explanatory variables that covaried with the coast–inland gradient were better predictors of the observed patterns of species richness and abundance than the gradient itself, we replaced distance to coast in the relevant optimal models with the excluded variables to see whether this improved the fit. The data used in the statistical analyses are available in Appendix S4.

## 3 RESULTS

We collected 4,077 oak‐associated beetle individuals from 205 species, of which the generalists were by far the most numerous and species‐rich group (Table 2).

**Table 2:** Summary statistics for all response variables measured in this study

Response variable (Explanation)	Species richness		Abundance	
	Mean (min–max)	Total	Mean (min–max)	Total
All species (All oak‐associated species)	32.7 (18–55)	205	127.4 (36–451)	4077
Mainly oak species (Species mainly occurring in oak)	4.4 (1–9)	25	17.4 (1–73)	557
Broadleaf species (Species occurring only in oak and broadleaved trees)	7.8 (3–15)	55	17.8 (4–70)	571
Generalists (Species occurring in oak and coniferous trees)	20.5 (11–36)	125	92.2 (22–432)	2949

Only oak‐associated beetles are included.

Min, minimum; max, maximum.

### 3.1 Environmental correlates with the coast–inland gradient

Only climate and landscape‐scale variables were correlated with distance from the coast (Table 3). The coastal historically first‐logged areas were warmer and dryer, had less area of old forest, and a greater forest volume per hectare than the inland sites (Table 3). There was also a close to significant trend with more forest cover and hollow oaks inland (Table 3).

**Table 3:** Pearson's correlation coefficients between selected continuous variables at different scales and the shortest distance to the coast (km) (*df* = 30 for all tests)

Variables	corr.	*p*‐Value
Tree variables		
Circumference	−0.012	0.948
Local scale		
Forest density	0.282	0.119
Deadwood	0.075	0.684
Number of oaks	0.135	0.462
Hollow oaks	0.337	0.059
Landscape scale		
Forest cover	0.347	0.052
Old forest	0.701	<**0.001**
Forest volume	−0.600	<**0.001**
Deciduous forest	−0.227	0.211
Favorable habitat	−0.019	0.916
Climate		
Precipitation	0.482	**0.005**
Temperature	−0.773	<**0.001**

The local scale was the surrounding landscape in a 42 × 42 m area centered on the hollow oak, whereas variables at the landscape scale were measured within a 2 km radius of that tree (see Table 1 for further details). Bold *p*‐values indicate significant variables.

### 3.2 Determinants of species richness and abundance

The total species richness increased with distance from the coast and was positively affected by tree circumference and the cover of deciduous forest in the landscape (Table 4). Species richness of “generalists” and “broadleaf species” followed a similar pattern, being positively related to distance from the coast and with the cover of deciduous forest in the landscape. In contrast, species mainly occurring in oak only responded to tree circumference (Table 4).

**Table 4:** Determinants of saproxylic beetle species richness derived from the optimum generalized linear Poisson models and negative binomial models (for the all beetles and oak generalist dataset due to overdispersion)

Response variable	*p*‐Value	Disp.	Predictor variable	Est.	*SE*	*z*‐Value	*p*‐Value
All species	**0.011**	1.204	Intercept	3.014	0.143	21.050	<**0.001**
			Circumference	0.001	0.000	1.964	**0.050**
			Distance	0.008	0.003	2.465	**0.014**
			Deciduous area	0.001	0.000	2.405	**0.016**
Generalists	0.057	1.149	Intercept	2.741	0.132	20.831	<**0.001**
			Distance	0.010	0.004	2.278	**0.023**
			Deciduous area	0.001	0.001	1.569	0.117
Broadleaf species	**0.012**	0.759	Intercept	1.650	0.160	10.301	<**0.001**
			Distance	0.013	0.005	2.435	**0.015**
			Deciduous area	0.001	0.001	2.270	**0.023**
Mainly oak species	0.054	1.111	Intercept	1.074	0.227	4.737	<**0.001**
			Circumference	0.002	0.001	1.972	**0.049**

We used backward elimination with AIC as the selection criterion, and the optimal models were tested against null models in analyses of deviance (for Poisson GLMs) or log‐likelihood tests (for negative binomial GLMs). The dispersion parameter (Disp.) of the model is shown and the *p*‐value from the tests against null models. Bold *p*‐values indicate significant predictor variables.

Overall abundance also increased with distance from the coast, but this pattern was not significant when analyzing the “mainly oak species,” the “broadleaf species” or the “generalists” separately (Table 5). Tree characteristics and local variables were most important in determining abundance, with all groups except the “broadleaf species” being positively affected by tree circumference and negatively affected by low and middle tree forms. The “broadleaf species” only responded to the local abundance of hollow oaks (Tables 5). The total oak‐associated beetle abundance and the abundance of the “mainly oak species” were also negatively influenced by local forest density, as indexed by stand basal area.

**Table 5:** Determinants of saproxylic beetle abundance present in the optimum negative binomial generalized linear models

Response variable	*p*‐Value	Disp.	Predictor variable	Est.	*SE*	*z*‐Value	*p*‐Value
All species	<**0.001**	1.212	Intercept	4.441	0.213	20.803	<**0.001**
			Circumference	0.002	0.001	3.367	**0.001**
			Distance	0.013	0.005	2.678	**0.007**
			Forest density	−0.015	0.008	−2.007	**0.045**
			Tree form low	−0.850	0.165	−5.141	<**0.001**
			Tree form middle	−0.587	0.141	−4.175	<**0.001**
Generalists	**0.001**	0.979	Intercept	3.851	0.227	16.962	<**0.001**
			Circumference	0.002	0.001	2.217	**0.027**
			Tree form low	−0.826	0.224	−3.691	<**0.001**
			Tree form middle	−0.639	0.192	−3.329	**0.001**
Broadleaf species	**0.010**	1.025	Intercept	2.517	0.200	12.575	<**0.001**
			Tree form low	−0.344	0.262	−1.314	0.189
			Tree form middle	0.348	0.216	1.609	0.108
			Hollow oaks	0.119	0.050	2.398	**0.017**
Mainly oak species	<**0.001**	1.041	Intercept	3.39	0.493	6.87	<**0.001**
			Circumference	0.004	0.001	2.526	**0.012**
			Tree form low	−1.607	0.384	−4.189	<**0.001**
			Tree form middle	−1.226	0.325	−3.770	<**0.001**
			Forest density	−0.034	0.017	−1.990	**0.047**

We used backward elimination with AIC as the selection criterion, and the optimal models were tested against null models in log‐likelihood tests. The dispersion parameter (Disp.) of the model is shown and the *p*‐value from the tests against null models. Bold *p*‐values indicate significant predictor variables.

Models fitted with the excluded collinear predictor variables were weaker, with no significant effects of the predictors (forest cover, forest volume, old forest, and temperature). Overall, distance to coast had the higher explanatory power for the observed patterns of species richness and abundance.

## 4 DISCUSSION

In this study, we hypothesized that beetle species richness and abundances should be highest inland as a result of later, lower intensity, historical logging compared with that in coastal areas. Our finding that total species richness and overall abundance increased inland supports this hypothesis, although the most specialized species for which we expected a clear response, did not respond to the coast–inland gradient. Present environmental conditions modified the beetle abundances at local and tree scale, whereas beetle species richness was affected at tree and landscape scales.

### 4.1 Are the effects of historical logging real?

Logging history in Southern Norway is not georeferenced, and thus, we used distance to the coast as a proxy for past logging. Several variables—climate, area of old forest, and volume—also change systematically along this gradient (see Table 3). Their influences cannot be clearly separated from those of historical logging, but if the observed species’ responses were due solely to climate, we would expect highest species richness along the coast where temperatures were high and precipitation low (Gough et al., 2015; Müller et al., 2015). This is opposite to the observed pattern. The forest structure changed along the gradient, with more extensive old forest and lower total forest volume inland than along the coast. When we replaced distance to coast with these variables in our models, however, no relationship was found with beetle species richness or abundances. As a hollow oak's distance to the coast, in itself, should not promote species richness, we therefore believe the most likely explanation of the observed pattern is a response to the historical logging intensity and duration.

The suggested negative effect of logging on saproxylic species richness fits well with other data (Gossner et al., 2013; Müller, Hothorn, & Pretzsch, 2007; Paillet et al., 2010; Siitonen, 2001). Intensive forest management in Finland has already led to the extinction of over a hundred forest‐dwelling species, but an extinction debt is probably still present in the northeastern inland areas where intensive forestry only started after World War II (Hanski & Ovaskainen, 2002; Kouki, Hyvarinen, Lappalainen, Martikainen, & Simila, 2012).

### 4.2 Why do species mainly associated with oak not respond to historical logging?

The species mainly associated with oak in our study did not respond as expected along the coast–inland gradient representing historical logging. We acknowledge that the low number of species within this group and the difficulty with correct categorization of host tree specialization (some “mainly oak species” also use other tree species) might have interfered with our results. If present in higher numbers, true specialists might have shown a pattern similar to what found for the broadleaf species. However, assuming that our data represent a specialist response, it is also possible that local extinctions happened rapidly and that the current populations are already in equilibrium with their environment at all sites. Another possible explanation is climate. Several of our most specialized species are apparently restricted to the warmest parts of the oak region (Norwegian Biodiversity Information Centre, 2016). Gough et al. (2015) found that oak specialists responded negatively to summer precipitation and positively to increased summer temperatures when studying a 700‐km climatic gradient across Sweden and Norway. Our inland sites should therefore be climatically less favorable. However, microclimate is also important for saproxylic beetles (Müller et al., 2015) and hollow oaks situated on southern slopes or the top of hills could experience higher temperatures than the average climate on a landscape scale that we used in our study. Finally, species within the strongest association to oak may respond heterogeneously to the gradient masking the predicted effect possibly present within a subset of the species.

### 4.3 From tree to landscape scale

In addition to the gradient of historical logging, we found that the environment influenced the oak‐associated beetle community at several spatial scales. The tree scale was important for species richness and abundance, whereas the local scale was only important for abundances and the landscape scale only for species richness (Tables 4 and 5). This indicates that different processes are important in determining abundance and species richness. Population sizes appear to be controlled by local resources, such as patch size and quality. For a species to maintain populations through time, however, larger areas of suitable habitat are needed and, in disturbed habitats, species could have died out because of increased isolation. If so, the greater species richness in deciduous forest at a landscape level makes sense, because deciduous forest provides more habitat in the form of host trees and deciduous deadwood.

Spatial patterns likely reflect differences in species’ dispersal biology (Bergman et al., 2012; Ranius, 2006). Many species living in hollow trees could be dispersal‐limited, given the stable and long‐lived habitats to which they are adapted (Nilsson & Baranowski, 1997; Ranius, 2006; Ranius & Hedin, 2001). Detailed studies of saproxylic beetles in hollow oaks indicate that their spatial responses to the surrounding environment vary at a range of scales (52 m to ≥5,000 m: Bergman et al., 2012, 135–2,800 m: Ranius, Johansson, & Fahrig, 2011) depending on species. In particular, the beetle species richness was best explained by oak density on a 2.3‐km scale (Bergman et al., 2012). This is a similar scale of response to the 2‐km landscape scale that we used, to which overall beetle species richness and the broadleaf species responded. Because the broadleaf species also can use other deciduous host trees, the positive effects of deciduous forest in the landscape could partly offset the negative effects of historical logging in areas where deciduous trees are prevalent.

At the tree level, the positive relationship between circumference and both species richness and abundances accords with previous studies (Buse, Entling, Ranius, & Assmann, 2016; Pilskog, Birkemoe, Framstad, & Sverdrup‐Thygeson, 2016; Ranius & Jansson, 2000; Sverdrup‐Thygeson et al., 2010). At this scale, circumference can be viewed as a proxy for patch size (Pilskog et al., 2016), often being associated with more wood mold and greater architectural diversity, and therefore an increasing number of available niches (Siitonen & Ranius, 2015). At the local scale, the observed negative relationship between forest density and beetle abundance fits well with previous studies showing that openness or limited regrowth around hollow oaks positively influences abundance (Gough, Birkemoe, & Sverdrup‐Thygeson, 2014; Ranius & Jansson, 2000; Widerberg, Ranius, Drobyshev, Nilsson, & Lindbladh, 2012). Lower forest density means less shade, increased insolation, and higher temperatures, likely to favor saproxylic beetles (Müller et al., 2015; Widerberg et al., 2012).

Wide‐branched solitary trees in agricultural landscapes typically have a low tree form which has been associated with high beetle abundance (Pilskog et al., 2016). This was not found in our study. As we focused on hollow oaks in forests or in the transition zone between agricultural landscapes and forests (seminatural landscapes), tree growth is typically tall and the growth form variable may represent current or historical environmental conditions not measured in our study.

### 4.4 Do the beetle communities in hollow oaks have an extinction debt?

Although our knowledge of historical habitat density for species in hollow oaks in most of Europe is limited, there is growing evidence that veteran trees and old‐growth forest are harboring extinction debts (Berglund & Jonsson, 2005; Sverdrup‐Thygeson, Gustafsson, et al., 2014). For example, occurrence of red‐listed lichen and fungus species on old oaks in Sweden was best explained by including the early 19th‐century oak density prior to large‐scale logging, indicating a probable extinction debt (Ranius, Eliasson, & Johansson, 2008). Buse (2012) found that saproxylic flightless weevils were absent from forests younger than 200 years and that their occurrence was explained by historical habitat density, but not current woodland size. Moreover, Nilsson and Baranowski (1997) found lower species richness of beetles in hollow trees in stands that had been managed 50–100 year ago, than in nearly primeval stands, suggesting slow recolonization.

As hollow oaks can last for centuries, it is possible that those in our study were colonized by beetles in the past when there was greater connectivity between oaks. Beetle populations living in hollow oaks can remain for decades, potentially even centuries (Hedin, Ranius, Nilsson, & Smith, 2008; Ranius & Hedin, 2001). Thus, the beetle populations in our regions may not be in equilibrium with their current surroundings (Ranius, 2002), in particular in areas with the most recent changes. If the isolation of hollow oaks inland and along the coast is similar today, the current difference in species richness could reflect an extinction debt in inland beetle communities. Our data show a close‐to‐significant (*p* = 0.059) increase in local hollow oak densities from coast to inland, but our variable “favorable habitat” based on relevant habitat amount at the landscape scale, did not vary accordingly. Thus, an extinction debt in the inland beetle communities may potentially contribute to explain the observed patterns.

## 5 CONCLUSIONS

Large old trees are disappearing globally at a faster rate than new ones are being recruited (Gibbons et al., 2008; Lindenmayer et al., 2014), and our results demonstrate the importance of including both habitat history, spanning several centuries, and current spatial occurrence when aiming to understand community dynamics in these long‐lasting habitats. We found population sizes to respond to local conditions of the tree and the close surroundings, but larger areas are necessary to maintain species richness through time. The echoes of the past also carry another important message: Actions taken today can affect species in hollow oaks far into the future. The good news is that the slow response of these species gives us time to improve their habitats and hopefully save those in decline. In Norway, we can expect the highest species richness inland, and local and national history might help predicting where the most valuable oaks could be in other countries.

## CONFLICT OF INTEREST

The authors declare that they have no competing interests.

## AUTHORS’ CONTRIBUTIONS

HP, AST, and TB developed the idea and design of the study. HP carried out the fieldwork, the statistical analyses, and wrote the manuscript drafts. ME helped with the selection of statistical analyses and interpretation. AST, TB, ME, and EF read and commented on manuscript drafts, and all authors commented on and approved the final manuscript.

## AVAILABILITY OF DATA AND MATERIALS

Appendix S4 with the data sets supporting the results of this article are available in the Dryad Digital Repository (https://doi.org/10.5061/dryad.674r3nf).
