# Supplementary material for: Long‐lasting effects of logging on beetles in hollow oaks
Source: Ecol Evol. 2018 Sep 27;8(20):10126–37. doi: 10.1002/ece3.4486 (PMC6206182; doi:10.1002/ece3.4486)
Supplement: Supplementary file 1 [file ECE3-8-10126-s001.docx]

# Supplementary material

Pilskog, H. E., Sverdrup-Thygeson, A., Evju, M., Framstad, E. and Birkemoe, T. Long-lasting effects of logging on beetles in hollow oaks.

# Appendix S1

**The logging of oak in southern Norway**

Historically, oak has been important to humans for centuries for building houses, ships, furniture, tanning, firewood and animal food. In Norway, timber export and log-floating has been known from around a thousand years ago (Vogt 1886, Central Bureau of Statistics of Norway 1977). Although there are records of timber trade with the Netherlands, England, Scotland and Denmark from the 13^th^ century and onwards (Vogt 1886, Vevstad 1998, Daly 2007), the largest exploitation of oaks in Norway happened after the waterwheel-driven gate saw was introduced and became common in rivers in the 1520s. At this time, there were large oak forests growing along the coastline of southern Norway (Vogt 1886, Vevstad 1998), and Scots and Dutch merchants came by ship and bought oak timber directly from farmers on the coast (Vevstad 1998, Moore 2010). Oak was especially important for building ships, but many places in Western Europe oaks were scarce or situated inland (Vevstad 1998, Moore 2010).

From around 1580 the timber trade could be characterized as large scale, with oak as the most sought-after timber and Holland one of its main destinations (Moore 2010), although the best oaks in general were reserved for the Danish-Norwegian king (Vevstad 1998). By the 1630s, many places along the coast lacked suitable oak timber (Tvethe 1852), and by the mid-17th century it was difficult to supply the Dutch with large oaks, causing them to switch their purchasing to the Baltic and Russia (Moore 2010). One can assume that, in general, logging of oak occurred inland from the mid-17th century onwards as the coastal oak forests in southern Norway became depleted, ending of the oak-logging boom there (Moore 2010). As oak became scarce, logging for pine and spruce intensified, progressively replacing oak as the most important timber tree (Vevstad 1998). The large coastal oak forests were gone and oak never regained its dominant role in the logging industry. Still oak timber was highly valued until the late-19^th^ century, before other shipbuilding materials became available. Because long-distance timber transport depended on water, the most attractive inland forests were those near large rivers on which logs could be floated. Throughout the 18^th^ and 19^th^ centuries the network of such rivers expanded and many rivers were modified to facilitate the timber transport. Log-floating remained the main mode of timber transport until the 1950s (Central Bureau of Statistics of Norway 1977). The coastal forests in the Larvik and Agder regions of our study have also been under more continuous land-use pressure than the inland forests as a result of the coastal development of cities and larger settlements. Logging to meet the demand for firewood and charcoal production for the metallurgical industries, along with grazing and collecting bark for tanning, all created constant pressure on forest resources.

Unfortunately, there are no good estimates of the extent of the original 16^th^ century oak forests or how much oak was logged, as there are no complete log-floating statistics prior to the 1870s (Central Bureau of Statistics of Norway 1977). Nevertheless, fragmentary records from customs accounts, the number of saws and forest registrations clearly show oak being heavily exploited from 1520 through to the 1600s (Tvethe 1852, Central Bureau of Statistics of Norway 1977), with little oak suitable for logging left by the end of the 17^th^ century (Fryjordet 1968, Moore 2010). It has been suggested that the export of oak in the period 1520−1630 increased 50-fold (Central Bureau of Statistics of Norway 1977). According to Moore (2010 and references therein), the Dutch imported 300 000 − 375 000 m^3^ timber annually (including pine) from Norway in the 17^th^ century, with an estimated 25 000 m^3^ of high quality oak being needed yearly in the 18^th^ century to maintain the Danish-Norwegian fleet (Vevstad 1995), despite there being a shortage of oak both in Norway and most of Western Europe at the time. The limited sawmill records seldom differentiate between oak and other trees after the 17^th^ century and only list the total quantum sawed in specific rivers, hence making it difficult to draw general trends about the oak quantum logged in this period. Throughout the 18^th^ and 19^th^ centuries Norway continued to be an international timber-producer nation with pine and spruce as the main products (Central Bureau of Statistics of Norway 1977, Hutchison 2012), although from the early 1900s onwards the value of oak was low.

**References**

Central Bureau of Statistics of Norway 1977. Timber Floating 1871-1975. Statistiske analyser. p 79.

Daly, A. 2007. Timber, Trade and Tree-rings. A dendrochronological analysis of structural oak timber in Northern Europe, c. AD 1000 to c. AD 1650. PhD thesis. University of Southern Denmark, p 270.

Fryjordet, T. 1968. Generalforstamtet 1739-1746. – Norsk skogbruksmuseum.

Hutchison, R. 2012. The Norwegian and Baltic timber trade to Britain 1780–1835 and its interconnections. – Scand. J. Hist. 37: 578-599.

Moore, J. W. 2010. ‘Amsterdam is Standing on Norway’ Part II: The Global North Atlantic in the Ecological Revolution of the Long Seventeenth Century. – J. Agrar. Change 10: 188-227.

Tvethe, M. B. 1852. Bidrag til Kundskab om Skovenes Tilstand i det 17de Aarhundrede. Norske Samlinger. Feilberg & Landmark, pp. 112-119 and pp. 592-608.

Vevstad, A. 1995. Skogen i Aust-Agder frå Skagerrak til fjellet. – Aust Agder Skogselskap.

Vevstad, A. 1998. Agderskog, Agder skogeierlag 1948-1998 og om skog og skogbruk på Agder gjennom tidene. – Agder Skogeigarlag.

Vogt, L. J. 1886. Om Norges Udførsel af Trælast i ældre Tider. Historisk Tidsskrift. Den Norske Historiske Forening, pp. 81-120 and 273-384.

# Appendix S2

Saproxylic beetle species and oak association

**Table A1.** The saproxylic beetle species and categorizations used in this study, along with the number of individuals collected. The categorization of these species and their oak associations was based on Dahlberg and Stokland (2004) (The Saproxylic Database) where species are listed as ‘occurring’ in or ‘preferring’ host tree species. We defined ‘mainly oak’ species as species noted as ‘preferring’ oak or only occurred on oak in the database; ‘broadleaf species’ as species occurring only on broadleaved trees (including oak); ‘generalists’ as species occurring on both broadleaf and coniferous trees (including oak); and ‘not oak’ for species not occurring on oak. The last group was not included in the analyses. The saproxylics includ both facultative and obligate saproxylic species. Species living in dead wood may change host tree depending on tree species availability within an area. Information status of species occurrence may also vary geographically. Hence, the species may be categorized differently between countries or if consulting different sources.

| **Family** | **Species** | **Oak association** | **Individuals** |
| --- | --- | --- | --- |
| Aderidae | *Euglenes pygmaeus* | mainly oak | 3 |
| Anthribidae | *Anthribus nebulosus* | generalist | 1 |
| Buprestidae | *Anthaxia quadripunctata* | not oak | 1 |
| Cantharidae | *Malthinus flaveolus* | generalist | 1 |
|  | *Malthinus frontalis* | not oak | 10 |
|  | *Malthodes brevicollis* | not oak | 4 |
|  | *Malthodes crassicornis* | not oak | 3 |
|  | *Malthodes fibulatus* | not oak | 2 |
|  | *Malthodes fuscus* | not oak | 3 |
|  | *Malthodes guttifer* | not oak | 17 |
|  | *Malthodes marginatus* | not oak | 6 |
|  | *Malthodes mysticus* | not oak | 1 |
|  | *Malthodes spathifer* | broadleaf | 1 |
| Carabidae | *Dromius agilis* | generalist | 12 |
|  | *Dromius fenestratus* | generalist | 2 |
|  | *Dromius quadrimaculatus* | mainly oak | 3 |
|  | *Platynus assimilis* | not oak | 7 |
| Cerambycidae | *Alosterna tabacicolor* | mainly oak | 10 |
|  | *Anastrangalia sanguinolenta* | not oak | 1 |
|  | *Leiopus nebulosus* | mainly oak | 4 |
|  | *Molorchus minor* | not oak | 1 |
|  | *Oxymirus cursor* | generalist | 1 |
|  | *Phymatodes testaceus* | mainly oak | 13 |
|  | *Rhagium inquisitor* | generalist | 1 |
|  | *Rhagium mordax* | generalist | 7 |
|  | *Stenurella melanura* | generalist | 2 |
|  | *Stictoleptura maculicornis* | generalist | 2 |
|  | *Tetropium fuscum* | not oak | 1 |
|  | *Tetrops praeusta* | not oak | 1 |
| Cerylonidae | *Cerylon fagi* | broadleaf | 1 |
|  | *Cerylon ferrugineum* | generalist | 23 |
|  | *Cerylon histeroides* | generalist | 12 |
| Ciidae | *Cis bidentatus* | generalist | 2 |
|  | *Cis boleti* | broadleaf | 12 |
|  | *Cis castaneus* | broadleaf | 2 |
|  | *Cis comptus* | generalist | 1 |
|  | *Cis festivus* | broadleaf | 16 |
|  | *Cis jacquemarti* | generalist | 1 |
|  | *Cis nitidus* | generalist | 1 |
|  | *Cis punctulatus* | not oak | 1 |
|  | *Cis submicans* | generalist | 11 |
|  | *Cis vestitus* | broadleaf | 1 |
|  | *Cis villosulus* | not oak | 3 |
|  | *Ennearthron cornutum* | generalist | 12 |
|  | *Octotemnus glabriculus* | broadleaf | 2 |
|  | *Orthocis alni* | generalist | 21 |
|  | *Sulcacis nitidus* | generalist | 2 |
| Cleridae | *Thanasimus formicarius* | generalist | 2 |
|  | *Tillus elongatus* | generalist | 1 |
| Coccinellidae | *Anatis ocellata* | not oak | 1 |
|  | *Aphidecta obliterata* | generalist | 2 |
|  | *Myrrha octodecimguttata* | not oak | 1 |
|  | *Scymnus suturalis* | not oak | 1 |
| Corylophidae | *Orthoperus rogeri* | not oak | 3 |
|  | *Sericoderus lateralis* | not oak | 1 |
| Cryptophagidae | *Atomaria diluta* | not oak | 3 |
|  | *Atomaria morio* | generalist | 1 |
|  | *Atomaria ornata* | not oak | 1 |
|  | *Cryptophagus dentatus* | broadleaf | 32 |
|  | *Cryptophagus dorsalis* | not oak | 18 |
|  | *Cryptophagus lapponicus* | not oak | 1 |
|  | *Cryptophagus micaceus* | not oak | 62 |
|  | *Cryptophagus parallelus* | not oak | 1 |
|  | *Cryptophagus scanicus* | generalist | 13 |
|  | *Cryptophagus setulosus* | broadleaf | 1 |
|  | *Micrambe abietis* | not oak | 5 |
|  | *Pteryngium crenatum* | not oak | 1 |
| Curculionidae | *Cryphalus asperatus* | not oak | 3 |
|  | *Crypturgus hispidulus* | not oak | 6 |
|  | *Dryocoetes alni* | not oak | 13 |
|  | *Dryocoetes autographus* | not oak | 6 |
|  | *Dryocoetes villosus* | mainly oak | 16 |
|  | *Hylastes brunneus* | not oak | 1 |
|  | *Hylastes cunicularius* | not oak | 5 |
|  | *Hylastes opacus* | not oak | 1 |
|  | *Hylesinus crenatus* | broadleaf | 2 |
|  | *Hylobius abietis* | generalist | 5 |
|  | *Kyklioacalles roboris* | not oak | 8 |
|  | *Magdalis armigera* | not oak | 1 |
|  | *Phloeophagus lignarius* | broadleaf | 1 |
|  | *Phloeotribus spinulosus* | not oak | 4 |
|  | *Pissodes pini* | not oak | 1 |
|  | *Pityogenes bidentatus* | not oak | 2 |
|  | *Pityogenes chalcographus* | not oak | 15 |
|  | *Pityophthorus lichtensteinii* | not oak | 19 |
|  | *Pityophthorus micrographus* | not oak | 1 |
|  | *Polydrusus cervinus* | not oak | 2 |
|  | *Polydrusus tereticollis* | not oak | 2 |
|  | *Polygraphus poligraphus* | not oak | 1 |
|  | *Rhyncolus ater* | generalist | 76 |
|  | *Rhyncolus elongatus* | not oak | 3 |
|  | *Rhyncolus sculpturatus* | generalist | 1 |
|  | *Scolytus intricatus* | mainly oak | 12 |
|  | *Strophosoma capitatum* | generalist | 36 |
|  | *Trypodendron domesticum* | broadleaf | 3 |
|  | *Trypodendron lineatum* | not oak | 3 |
|  | *Trypodendron signatum* | broadleaf | 14 |
|  | *Xyleborinus saxeseni* | broadleaf | 5 |
|  | *Xyleborus dispar* | mainly oak | 3 |
| Dasytidae | *Aplocnemus nigricornis* | generalist | 1 |
|  | *Dasytes caeruleus* | generalist | 5 |
|  | *Dasytes niger* | generalist | 11 |
|  | *Dasytes plumbeus* | broadleaf | 18 |
| Dermestidae | *Anthrenus museorum* | not oak | 11 |
|  | *Attagenus pellio* | broadleaf | 3 |
|  | *Ctesias serra* | broadleaf | 3 |
|  | *Megatoma undata* | generalist | 5 |
| Elateridae | *Ampedus balteatus* | generalist | 274 |
|  | *Ampedus hjorti* | mainly oak | 31 |
|  | *Ampedus nigrinus* | generalist | 38 |
|  | *Ampedus nigroflavus* | broadleaf | 1 |
|  | *Ampedus pomorum* | generalist | 3 |
|  | *Athous subfuscus* | not oak | 167 |
|  | *Cardiophorus ruficollis* | generalist | 1 |
|  | *Crepidophorus mutilatus* | broadleaf | 1 |
|  | *Denticollis linearis* | generalist | 10 |
|  | *Hypoganus inunctus* | broadleaf | 1 |
|  | *Melanotus castanipes* | not oak | 109 |
|  | *Sericus brunneus* | not oak | 1 |
| Endomychidae | *Endomychus coccineus* | broadleaf | 3 |
|  | *Leiestes seminiger* | not oak | 2 |
|  | *Mycetina cruciata* | generalist | 2 |
| Erotylidae | *Dacne bipustulata* | broadleaf | 8 |
|  | *Triplax aenea* | not oak | 2 |
|  | *Triplax russica* | broadleaf | 21 |
| Eucnemidae | *Eucnemis capucina* | not oak | 4 |
|  | *Hylis procerulus* | not oak | 1 |
|  | *Microrhagus lepidus* | not oak | 3 |
|  | *Microrhagus pygmaeus* | generalist | 2 |
|  | *Xylophilus corticalis* | generalist | 35 |
| Histeridae | *Gnathoncus buyssoni* | not oak | 31 |
|  | *Plegaderus caesus* | broadleaf | 4 |
|  | *Plegaderus vulneratus* | not oak | 1 |
| Latridiidae | *Cartodere constricta* | generalist | 1 |
|  | *Cartodere nodifer* | generalist | 1 |
|  | *Corticaria longicollis* | generalist | 5 |
|  | *Corticarina fuscula* | not oak | 1 |
|  | *Corticarina obfuscata* | not oak | 1 |
|  | *Corticarina similata* | not oak | 2 |
|  | *Cortinicara gibbosa* | not oak | 13 |
|  | *Dienerella vincenti* | generalist | 3 |
|  | *Enicmus fungicola* | generalist | 3 |
|  | *Enicmus rugosus* | generalist | 70 |
|  | *Enicmus testaceus* | generalist | 29 |
|  | *Enicmus transversus* | not oak | 1 |
|  | *Latridius consimilis* | generalist | 2 |
|  | *Latridius gemellatus* | generalist | 1 |
|  | *Latridius hirtus* | generalist | 14 |
|  | *Latridius minutus* | generalist | 2 |
|  | *Stephostethus alternans* | not oak | 1 |
|  | *Stephostethus rugicollis* | not oak | 3 |
| Leiodidae | *Agathidium badium* | generalist | 3 |
|  | *Agathidium confusum* | generalist | 4 |
|  | *Agathidium mandibulare* | generalist | 1 |
|  | *Agathidium rotundatum* | generalist | 1 |
|  | *Agathidium seminulum* | generalist | 24 |
|  | *Agathidium varians* | generalist | 5 |
|  | *Anisotoma castanea* | not oak | 2 |
|  | *Anisotoma humeralis* | generalist | 66 |
|  | *Anisotoma orbicularis* | generalist | 1 |
|  | *Nemadus colonoides* | mainly oak | 6 |
| Lucanidae | *Platycerus caraboides* | broadleaf | 1 |
|  | *Sinodendron cylindricum* | broadleaf | 4 |
| Lycidae | *Dictyoptera aurora* | not oak | 6 |
|  | *Pyropterus nigroruber* | generalist | 2 |
| Lymexylidae | *Elateroides dermestoides* | generalist | 4 |
|  | *Lymexylon navale* | mainly oak | 1 |
| Melandryidae | *Abdera flexuosa* | not oak | 1 |
|  | *Conopalpus testaceus* | broadleaf | 6 |
|  | *Hypulus quercinus* | mainly oak | 1 |
|  | *Orchesia micans* | generalist | 5 |
|  | *Orchesia undulata* | broadleaf | 18 |
|  | *Phloiotrya rufipes* | broadleaf | 3 |
|  | *Serropalpus barbatus* | not oak | 3 |
|  | *Xylita laevigata* | not oak | 2 |
| Monotomidae | *Rhizophagus bipustulatus* | mainly oak | 30 |
|  | *Rhizophagus cribratus* | mainly oak | 19 |
|  | *Rhizophagus dispar* | generalist | 14 |
|  | *Rhizophagus fenestralis* | generalist | 3 |
|  | *Rhizophagus ferrugineus* | not oak | 1 |
|  | *Rhizophagus nitidulus* | generalist | 1 |
|  | *Rhizophagus parallelocollis* | not oak | 2 |
| Mordellidae | *Mordella holomelaena* | not oak | 2 |
|  | *Tomoxia bucephala* | generalist | 1 |
| Mycetophagidae | *Litargus connexus* | generalist | 3 |
|  | *Mycetophagus atomarius* | broadleaf | 1 |
|  | *Mycetophagus piceus* | mainly oak | 8 |
|  | *Mycetophagus populi* | not oak | 2 |
|  | *Triphyllus bicolor* | broadleaf | 9 |
| Nitidulidae | *Cryptarcha strigata* | broadleaf | 25 |
|  | *Cryptarcha undata* | broadleaf | 15 |
|  | *Cychramus luteus* | not oak | 10 |
|  | *Cychramus variegatus* | generalist | 1 |
|  | *Epuraea abietina* | generalist | 1 |
|  | *Epuraea aestiva* | not oak | 6 |
|  | *Epuraea guttata* | mainly oak | 1 |
|  | *Epuraea marseuli* | generalist | 2 |
|  | *Epuraea neglecta* | broadleaf | 2 |
|  | *Epuraea rufomarginata* | generalist | 3 |
|  | *Epuraea silacea* | not oak | 1 |
|  | *Epuraea unicolor* | generalist | 1 |
|  | *Glischrochilus hortensis* | broadleaf | 72 |
|  | *Glischrochilus quadriguttatus* | broadleaf | 6 |
|  | *Ipidia binotata* | generalist | 2 |
|  | *Pityophagus ferrugineus* | not oak | 2 |
|  | *Soronia grisea* | broadleaf | 33 |
| Ptiliidae | *Pteryx suturalis* | generalist | 1 |
| Ptinidae | *Cacotemnus rufipes* | broadleaf | 2 |
|  | *Dorcatoma chrysomelina* | mainly oak | 111 |
|  | *Dorcatoma dresdensis* | generalist | 2 |
|  | *Dryophilus pusillus* | not oak | 3 |
|  | *Ernobius mollis* | not oak | 1 |
|  | *Grynobius planus* | broadleaf | 24 |
|  | *Hemicoelus canaliculatus* | broadleaf | 6 |
|  | *Ptilinus pectinicornis* | broadleaf | 3 |
|  | *Ptinomorphus imperialis* | broadleaf | 5 |
|  | *Ptinus fur* | generalist | 6 |
|  | *Ptinus rufipes* | mainly oak | 1 |
|  | *Ptinus subpillosus* | mainly oak | 172 |
|  | *Xestobium rufovillosum* | mainly oak | 70 |
| Salpingidae | *Salpingus planirostris* | broadleaf | 7 |
|  | *Salpingus ruficollis* | generalist | 10 |
|  | *Sphaeriestes castaneus* | not oak | 1 |
| Scarabaeidae | *Cetonia aurata* | not oak | 1 |
|  | *Trichius fasciatus* | broadleaf | 1 |
| Scirtidae | *Prionocyphon serricornis* | broadleaf | 1 |
| Scraptiidae | *Anaspis frontalis* | generalist | 6 |
|  | *Anaspis marginicollis* | not oak | 51 |
|  | *Anaspis rufilabris* | generalist | 64 |
|  | *Anaspis thoracica* | generalist | 4 |
| Silphidae | *Phosphuga atrata* | generalist | 1 |
| Silvanidae | *Silvanoprus fagi* | not oak | 2 |
| Sphindidae | *Aspidiphorus orbiculatus* | generalist | 3 |
| Staphylinidae | *Acidota crenata* | not oak | 18 |
|  | *Atheta amicula* | not oak | 1 |
|  | *Atheta corvina* | not oak | 2 |
|  | *Atheta crassicornis* | not oak | 6 |
|  | *Atheta euryptera* | not oak | 1 |
|  | *Atheta hypnorum* | not oak | 2 |
|  | *Atheta laticollis* | not oak | 1 |
|  | *Atheta picipes* | generalist | 1 |
|  | *Atheta sodalis* | not oak | 11 |
|  | *Atheta vaga* | generalist | 31 |
|  | *Atrecus affinis* | generalist | 2 |
|  | *Batrisodes venustus* | generalist | 5 |
|  | *Bibloporus bicolor* | generalist | 90 |
|  | *Bisnius fimetarius* | not oak | 2 |
|  | *Bryaxis puncticollis* | not oak | 1 |
|  | *Coprophilus striatulus* | not oak | 2 |
|  | *Dadobia immersa* | generalist | 7 |
|  | *Dexiogyia forticornis* | not oak | 8 |
|  | *Dinaraea aequata* | generalist | 1 |
|  | *Dropephylla ioptera* | generalist | 3 |
|  | *Euconnus claviger* | generalist | 3 |
|  | *Euplectus bescidicus* | not oak | 9 |
|  | *Euplectus karstenii* | generalist | 33 |
|  | *Euplectus mutator* | generalist | 7 |
|  | *Euplectus nanus* | generalist | 8 |
|  | *Euplectus piceus* | generalist | 16 |
|  | *Euplectus punctatus* | generalist | 13 |
|  | *Euryusa castanoptera* | not oak | 2 |
|  | *Gabrius splendidulus* | generalist | 21 |
|  | *Gyrophaena boleti* | not oak | 1 |
|  | *Gyrophaena joyioides* | not oak | 1 |
|  | *Hapalaraea pygmaea* | broadleaf | 7 |
|  | *Haploglossa gentilis* | mainly oak | 35 |
|  | *Haploglossa villosula* | generalist | 1484 |
|  | *Holobus apicatus* | generalist | 2 |
|  | *Holobus flavicornis* | not oak | 3 |
|  | *Ischnoglossa prolixa* | generalist | 8 |
|  | *Leptusa fumida* | generalist | 12 |
|  | *Leptusa pulchella* | generalist | 8 |
|  | *Leptusa ruficollis* | broadleaf | 56 |
|  | *Lordithon lunulatus* | generalist | 2 |
|  | *Mniusa incrassata* | not oak | 4 |
|  | *Mycetoporus lepidus* | not oak | 33 |
|  | *Neuraphes elongatulus* | not oak | 1 |
|  | *Neuraphes plicicollis* | not oak | 2 |
|  | *Omalium rugatum* | not oak | 1 |
|  | *Oxypoda arborea* | broadleaf | 68 |
|  | *Pella cognata* | generalist | 8 |
|  | *Pella funesta* | generalist | 1 |
|  | *Pella laticollis* | generalist | 4 |
|  | *Pella lugens* | generalist | 13 |
|  | *Philonthus succicola* | not oak | 5 |
|  | *Phloeocharis subtilissima* | generalist | 2 |
|  | *Phloeopora corticalis* | generalist | 3 |
|  | *Phloeopora testacea* | generalist | 5 |
|  | *Phloeostiba plana* | generalist | 1 |
|  | *Phyllodrepa melanocephala* | broadleaf | 1 |
|  | *Plectophloeus nitidus* | generalist | 2 |
|  | *Quedius brevicornis* | generalist | 6 |
|  | *Quedius brevis* | not oak | 1 |
|  | *Quedius cruentus* | broadleaf | 1 |
|  | *Quedius fuliginosus* | not oak | 1 |
|  | *Quedius maurus* | generalist | 6 |
|  | *Quedius mesomelinus* | broadleaf | 12 |
|  | *Quedius scitus* | mainly oak | 2 |
|  | *Quedius xanthopus* | generalist | 52 |
|  | *Scaphisoma agaricinum* | generalist | 15 |
|  | *Scaphisoma boreale* | not oak | 4 |
|  | *Scydmoraphes minutus* | generalist | 3 |
|  | *Sepedophilus littoreus* | generalist | 2 |
|  | *Sepedophilus testaceus* | generalist | 9 |
|  | *Stenichnus bicolor* | generalist | 5 |
|  | *Stenichnus godarti* | mainly oak | 2 |
|  | *Syntomium aeneum* | not oak | 1 |
|  | *Thamiaraea cinnamomea* | broadleaf | 3 |
|  | *Thamiaraea hospita* | mainly oak | 1 |
|  | *Xantholinus tricolor* | not oak | 1 |
| Tenebrionidae | *Mycetochara axillaris* | not oak | 5 |
|  | *Mycetochara flavipes* | broadleaf | 4 |
|  | *Mycetochara maura* | broadleaf | 16 |
|  | *Palorus depressus* | mainly oak | 2 |
|  | *Prionychus ater* | broadleaf | 3 |
|  | *Pseudocistela ceramboides* | generalist | 22 |
|  | *Uloma rufa* | not oak | 1 |
| Tetratomidae | *Hallomenus binotatus* | generalist | 5 |
|  | *Tetratoma ancora* | generalist | 1 |
| Trogossitidae | *Grynocharis oblonga* | generalist | 1 |
|  | *Thymalus limbatus* | generalist | 11 |
|  |  | **Total** | **4919** |

**References**

Dahlberg, A., Stokland. J. 2004. Substrate requirements of wood-inhabiting species - a synthesis and analysis of 3600 species. Skogsstyrelsen. Report 7- 04, pp. 75.

# Appendix S3

The habitat variable

We used records from the Norwegian database for habitats (Naturbase) (Norwegian Environment Agency 2015) and non-digitalized maps from Larvik to construct the habitat variable. This variable was measured at the landscape scale. From the Norwegian database for habitats we used ‘selected habitat type’ for hollow and large oaks (point registrations and polygons), and standing and lying deadwood (polygons). Point registrations of hollow deciduous trees were obtained from Complementary Hotspot Inventories. In addition, non-digitized woodland key habitats from Larvik were acquired by entering the center point of the polygons in ArcMap 10.2.2 and using the buffer function to make the polygons with their corresponding sizes. All polygon types and points registrations were checked for overlap in ArcMap, and only the largest overlapping values were included.

In contrast to the other polygons, the deadwood polygons from the Norwegian database for habitats had a defined number of trees per hectare (20-40 trees ha^-1^) (Baumann et al. 2001). We therefore converted the other records to this scale. Point registrations of trees were counted and the total was converted to a hectare measure using the definition of 30 trees ha^-1^. The large oak polygons generally included more habitat than old oaks, and we therefore used information from the database on number of oaks in each polygon and converted this number to create new polygons whose size was equivalent to 30 trees ha^-1^. For polygons with insufficient information, we used a conversion factor of 0.22, which corresponded to the average conversion factor used for the other hollow oak polygons. A conversion factor of 0.1 was used for the woodland key habitats in Larvik, as this conversion factor made the polygons more comparable to the other registrations, and more likely to reflect the definition of 30 trees ha^-1^. Lastly, all these converted records were added to the deadwood polygons to make one habitat variable reflecting area of favorable habitat at the 2-km scale around each studied hollow oak.

**References**

Baumann, C. et al. 2001. Miljøregistrering i skog - biologisk mangfold. Håndbok i registrering av livsmiljøer i skog. Hefte 3. Instruks for registrering 2001. – Skogforsk, NIJOS and Landbruksdepartementet.

Norwegian Environment Agency 2015. Norwegian database for habitats (Naturbase) -Norwegian Environment Agency (Accessed 30 September, 2015) <http://karteksport.miljodirektoratet.no/#page=tab1>
